# Polishing Plates

**Published:** 1862-02

**Authors:** George Beers

**Affiliations:** Montreal


Polishing Plates.—*By George Beers, Montreal.*—The importance of properly and efficiently polishing plates, and *finishing* a set of teeth, has never, in my opinion, been sufficiently considered. Many dentists think it a waste of time, but the time occupied in properly finishing a plate, is fully repaid the operator in the additional appreciation of the set by the patient, and the consciousness of the dentist, that he has faithfully performed every detail of his agreement. For though the dentist may make no verbal agreement to "polish the plate," he is *expected* to do it, as much as the artist is to finish a painting, a sculptor a sculpture, or a jeweller a piece of jewelry.

A set of teeth should be polished that it may be

"A theme of admiration and delight,"

out of the mouth as well as in it; the plate should properly reflect the image, and each lining make a miniature mirror in itself.

Patients who receive an unsufficiently polished set, frequently complain of paying for "half-done work"—that is, *if* they pay at all—and their ignorance of dentistry magnifies the fault sometimes to an alarming extent. Then, if it ever requires repairing, it may be taken to another dentist, who takes the time to polish it up, and the patient pays for the repaired set, with some such exclamation as, "Why, it looks better than when I first got it!" Thus by a desire of saving a little time such saving is *lost*—the operator is upbraided by his patient, and the four corners of the globe, if the latter visits them, hear the mournfully exaggerated tale.

There are some dentists who will polish a plate for a *dental exhibition*, as well as any jeweler, but when it is for a patient, their ardent desire of finishing it is remarkably lessened. One reason for this is, that they do not get a written diploma of merit from their patient, as they would from an exhibition, nor do they expect it will be so critically examined as in the latter; but here they are sadly mistaken, for the patient gives them a diploma of *demerit*, signed and sealed by his untiring tongue, and to give more force to his arguments may produce the set, embellishing (?) it with epithets where it might have been embellished with *emery and rouge* to a much more profitable degree.

There are a few little points previous to soldering which, if followed, will conduce to a more easy finishing of the plate; first, it is advisable to have the *dies* as smooth as possible, with no unnecessary projections that would deface it; secondly, in striking it up, care should be taken of dinging its surface with sharp hammers, or uneven strokes, and no hammer used but one quite rounded on its beating surface; thirdly, care should be taken to solder the set well, flowing the solder as evenly as possible.

After the set is soldered and quite cool, it should be boiled for a few minutes in sulphuric acid and water (about ʒ i. of S.O.$^3$ to ʒ v. of water) to dissolve the borax, and then it is ready for finishing. I commence by taking off and evening all superfluous solder, with the bur on my lathe; or by the use of scrapers and files I reduce it quite as well, but not so rapidly. There is nothing, however, like the bur on the lathe to trim the bottom of a lining, or the lining itself. After reducing or trimming the solder as I wish, I take a piece of the finest sand paper, and go over every part of the plate, taking care not to rub too hard or too long. To get in the little interstices of a plate, files of various shapes are used with great advantage. Some may think this sand-papering injurious to the plate; it is, of course, if used too much, like many other things, but it is taken for granted that the readers of the *Dental Journal* are not so stupidly devoid of a little common sense or judgment, as to think I mean them to rub away till they rub through the plate. After the use of sand-paper (it must be very fine), I take a blunt, round-pointed scraper—the point something of the shape of the neck of a plain artificial incisor—and scrape evenly and easily over every part of the plate. It must not be used so as to *scale* off the metal, but so that it will smoothen and burnish at the same time. I find this very efficient to insure a good polish, and if properly and carefully done, I will guarantee as fine a finished set as can be made. The edges of the plate should now be finished off neatly with a smooth file. I now take a cork wheel made to fit my lathe—of which wheels I use two, one pointed like a pyramid, the other like a common corundum wheel, and use it over every part of the plate with pumice-stone. Wherever the cork wheel can not get, I use a pointed piece of cork or cane with the hand, after which, I take a burnisher, and burnish every part of the plate, then follow with emery, giving the plate a thorough brushing.

Previous to using the emery, or pumice-stone, I would advise filling up all the interstices between the teeth and linings, to prevent the lodgment of food, etc., also to keep the pumice-stone or emery from getting in the spaces, which it is sometimes difficult to get out again. For this purpose some use amalgam, others gold or tin foil, and others osteo-plastic. The first takes too long to harden, and for various reasons is objectionable; the second cannot be made to stay in well without a waste of time in condensing and working it; the third has my preference, and to tell the truth, I think that's all it is good for. It hardens soon, is easily put in, and can be colored with a little rouge to the *exact* shade of the gum, if required. After thoroughly washing off the emery with soap and water, I use rouge, wet with alcohol or water. There are various powders and polishes for plate work, but of all I have tried, I find nothing to surpass good rouge. Prepared chalk, magnesia, "tripoli," and a dozen others have all been tried, but rouge still maintains its supremacy.

The above manner of polishing a plate is for gold or platinum, and in most parts for the vulcanite base also. But platinum requires a little different process for a *perfect* finish. The use of Scotch stone with pumice-stone, will smoothen platinum well. A correspondent of the *Chemical News* gives the following method of cleaning platinum. He says it may be cleaned very rapidly and perfectly by "gently rubbing upon the dirty metal a small lump of *sodium amalgam*. This should be rubbed on with a cloth, until the whole surface is brilliantly metallic; water is then applied, which oxidizes the sodium and allows the cohesion of the mercury to assert itself. The plate is now wiped off, and the platinum surface is left in admirable condition for the burnisher." It is said that the "wetted" metal does not suffer the least trace of amalgamation, and "even when foreign metals, such as lead, tin, zinc, silver, are purposely added to the sodium-amalgam, the platinum surface suffers no disintegration."

To polish a set of vulcanite, I use the method generally recommended, viz.: with different shaped files, sand-paper, pumice-stone, emery and rouge. The use of the cork-wheel and pumice-stone on the vulcanite is a certain method of taking out the scratches of the file or sand-paper.

In finishing off *partial* sets with clasps, the latter should be filed with a "worn-out" file inside and out, and the edge, or rims that touch the inside of the natural teeth, filed and burnished smoothly.

When finishing off a plate it is liable to accident from bending during the process of polishing, also from bristles of the brushes catching between the teeth or in the clasps, and dragging the plate out of the hand of the operator, and from various other accidents that should be guarded against. It is by no means pleasant to break a tooth or bend the plate just when it is polished, and I am sure that this will seldom occur if the operator keeps his wits about him, and be "canny" as the Scotch call it.

If a dentist can make a set to both *fit* and *look well*, he may be called a good mechanical dentist, but if he makes a set to fit well, and *not look well*, he may be called a good dentist, but he has careless taste, that has room for improvement.—*New York Dental Journal.*

